# Effective Tumor Annotation for Automated Diagnosis of Liver Cancer

**DOI:** 10.1109/JTEHM.2025.3576827

**Published:** 2025-06-05

**Authors:** Yi-Hsuan Chuang, Ja-Hwung Su, Tzu-Chieh Lin, Hue-Xin Cheng, Pin-Hao Shen, Jin-Ping Ou, Ding-Hong Han, Yi-Wen Liao, Yeong-Chyi Lee, Yu-Fan Cheng, Tzung-Pei Hong, Katherine Shu-Min Li, Yi Lu, Chih-Chi Wang

**Affiliations:** Liver Transplantation ProgramKaohsiung Chang Gung Memorial Hospital Niaosung District Kaohsiung 833401 Taiwan; Department of Diagnostic RadiologyKaohsiung Chang Gung Memorial Hospital Niaosung District Kaohsiung 833401 Taiwan; College of MedicineChang Gung University 56081 Taoyuan City 33302 Taiwan; Department of Computer Science and Information EngineeringNational University of Kaohsiung 63286 Kaohsiung 811726 Taiwan; Department of Computer Science and EngineeringNational Sun Yat-sen University 34874 Kaohsiung 804201 Taiwan; Department of Intelligent CommerceNational Kaohsiung University of Science and Technology 517768 Kaohsiung 824004 Taiwan; Department of Information ManagementCheng Shiu University 63330 Kaohsiung 833301 Taiwan; Liver Transplantation CenterKaohsiung Chang Gung Memorial Hospital Niaosung District Kaohsiung 833401 Taiwan; Department of SurgeryKaohsiung Chang Gung Memorial Hospital Niaosung District Kaohsiung 833401 Taiwan

**Keywords:** Liver cancer diagnosis, liver tumor segmentation, liver tumor measuring, liver tumor location, liver tumor recognition

## Abstract

In recent years, visual cancer information retrieval using Artificial Intelligence has been shown to be effective in diagnosis and treatment. Especially for a modern liver-cancer diagnosis system, the automated tumor annotation plays a crucial role. So-called tumor annotation refers to tagging the tumor in Biomedical images by computer vision technologies such as Deep Learning. After annotation, the tumor information such as tumor location, tumor size and tumor characteristics can be output into a clinical report. To this end, this paper proposes an effective approach that includes tumor segmentation, tumor location, tumor measuring, and tumor recognition to achieve high-quality tumor annotation, thereby assisting radiologists in efficiently making accurate diagnosis reports. For tumor segmentation, a Multi-Residual Attention Unet is proposed to alleviate problems of vanishing gradient and information diversity. For tumor location, an effective Multi-SeResUnet is proposed to partition the liver into 8 couinaud segments. Based on the partitioned segments, the tumor is located accurately. For tumor recognition, an effective multi-labeling classifier is used to recognize the tumor characteristics by the visual tumor features. For tumor measuring, a regression model is proposed to measure the tumor size. To reveal the effectiveness of individual methods, each method was evaluated on real datasets. The experimental results reveal that the proposed methods are more promising than the state-of-the-art methods in tumor segmentation, tumor measuring, tumor localization and tumor recognition. Specifically, the average tumor size error and the annotation accuracy are 0.432 cm and 91.6%, respectively, which suggest potential for reducing radiologists’ workload. In summary, this paper proposes an effective tumor annotation for an automated diagnosis support system. Clinical and Translational Impact Statement—The proposed methods have been evaluated and shown to significantly improve the efficiency and accuracy of liver tumor annotation, reducing the time required for radiologists to complete reports on tumor segmentation, liver partition, tumor measuring and tumor recognition. By integrating into existing clinical decision support systems, it has the potential to reduce diagnostic errors and treatment delays, thereby improving patient outcomes and clinical workflow.

## I. Introduction

With advances of Biomedical Information Retrieval (BIR), significant successes have been achieved over the years in diagnosis, treatment and prognosis. This enables a large increase of medical support systems, particularly for liver cancer [1]. For these needs, a number of previous works were devoted to CT (Computed Tomography) image recognition. Based on image recognition, the practical system for diagnosing CT liver-tumors can segment the tumors automatically. Then, the radiologist manually annotates the tumor information into a diagnosis report, such as tumor location, tumor size and tumor characteristics. This inefficient procedure motivates us to propose an advanced solution to assist the radiologists in efficiently making an effective diagnosis report [2], [3] for CT liver tumors.

**FIGURE 1.**
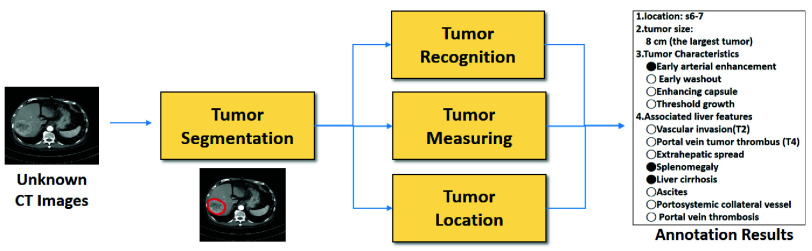
Scenario of proposed tumor annotation system.

Figure 1 shows the scenario of proposed tumor annotation system. In this diagram, the tumor segmentation serves as the foundational component for the next three elements, namely tumor recognition, tumor measuring and tumor location. Then, the annotation results will be generated according to these three elements. Note that, in this paper, the difference between tumor segmentation and tumor recognition lies in that the tumor segmentation refers to marking the tumor region, while the tumor recognition involves analyzing the tumor by visual features of the marked region.

In fact, implementing such annotation systems will encounter challenges in simultaneously performing effective tumor segmentation, tumor recognition, tumor measuring and tumor location to achieve more reliable diagnoses. In summary, the motivations and benefits of this paper can be folded as follows.

• In terms of tumor segmentation, traditional methods often suffer from information loss and uniform feature weighting. To address these issues, this paper proposes a Multi-Residual Attention U-Net (MRAU-Net), which integrates continuous residual connections along with channel-wise and pixel-wise attention mechanisms to enhance feature representation and improve segmentation performance.
• In terms of tumor location, the liver must first be partitioned into 8 regions (called liver partition in this paper), after which the tumor is localized within these specific regions. Actually, most related works have focused on liver partition using a single, highly complex model. In contrast, we propose multi-classifier framework, called Multi-SeResUnet, to decrease the computational complexity and enable accurate tumor localization within specific liver regions.
• For tumor measuring and tumor labeling, they have received limited attention in the literatures, as they are often considered less critical than tumor segmentation and liver partition. However, these two tasks are essential for the development of a fully functional medical support system in practice. To tackle this concern, the tumor size in this paper is calculated by the proposed regression model, which is based on the tumor segmentation. Additionally, tumor characteristics are effectively recognized through the use of a multi-labeling classifier.
• Regarding the clinical translational contribution, currently, this tumor annotation system is being tested by the radiologists at Chang Gung Memorial Hospital, Kaohsiung Branch, Taiwan. Preliminary feedbacks have been positive, as the system demonstrates the potential to significantly reduce the clinical workload associated with liver tumor assessment reporting. By streamlining the reporting process, it will help improve diagnostic efficiency and consistency in clinical practice. The average reporting time per case is expected to be reduced by at least 30%, thereby enabling radiologists to concentrate more on complex and high-priority diagnostic tasks. Additionally, it has the potential to reduce misjudgments and treatment delays. In the near future, the system is planned to be deployed across other branches of Chang Gung Memorial Hospital in Taiwan, further demonstrating its clinical applicability.

## II. Literature Review

The related works can be categorized into 3 classes, namely liver tumor segmentation, liver tumor recognition and liver partition. In the followings, these related works will be reviewed by classes.

### A. Liver Tumor Segmentation

Liver tumor segmentation has been a hot topic in the field of Bioinformatics due to rapid advances on Deep Learning. A number of forerunners proposed effective methods on this topic. In visual-based Deep Learning, Convolutional Neural Network (CNN) [5] is the backbone optimizing the features by a set of convolutions and gradient descents. Attributed to CNN’s excellent feature extraction capabilities, it has achieved successes in segmentation tasks. A fully convolutional neural network (FCN) [6], [7], [8] one of CNNs is efficient for object segmentation due to the end-to-end training. Next, Unet [9] is the most popular network for Biomedical image segmentation. Basically, the naïve Unet considers the horizontal information loss by concatenating the encoder and decoder. However, there remains another problem: vertical vanishing gradient. Here, so-called vertical vanishing gradient indicates the information loss while encoding and decoding. To attack this problem, ResUnet [10] added residual structures to the convolutions, reducing the vertical vanishing gradients. Also, Unet++ [11], [12] was extended to merge Unets of different depths as a global Unet. These sub-Unets share an encoder and each has its own decoder. In order to train them simultaneously, their losses are calculated together, and then the gradients are backpropagated to each other. In addition to residual-based Unet, attention gates [13], [14] were embedded into Unet, making the network focus on interested objects. The other recent type of Unets is DefED-Net [15] incorporating deformable convolutions and Ladder-ASPP (Atrous-Spatial-Pyramid-Pooling) into the Unet for scale granularity concerns.

### B. Liver Tumor Recognition

In this paper, tumor recognition is based on the segmented tumor. According to this concept, how to effectively recognize the tumor by feature analysis is the major aim. For example, Jiang et al. [16] detected microvascular invasion in hepatocellular carcinoma by comparing XGBoost and deep learning based on Radiomics features. Zhu et al. [17], and Shan et al. [18] adopted Radiomics features to predict early recurrence of hepatocellular carcinoma (HCC) after treatments. For microvascular invasion and outcome, Sun et al. [19] and Xu et al. [20] used Deep Learning and multivariate logistic regression to achieve the predictions for MRI and CT images, respectively. On the contrary, without microvascular invasion, Wei et al. [21] attempted to predict early recurrence of solitary HCC by MRI images. For early recurrence in HCC after hepatectomy, Zhao et al. [22] offered comparisons of clinical and imaging data for early recurrence and non-early recurrence. Later, Yan et al. [23] extracted the significant features from MRI images by VGG19 and compared 5 classifiers then. Tian et al. [24] provided a diagnosis support system tagging tumors with shape, contour, and intensity by a semi-supervised attention mechanism. Srinivasan et al. [25] aimed at the X-ray reporting by TCNet (Tag Classification Net) with Multi-head attention mechanism. Also, Endo et al. [26] projected visual and textual features on a space and retrieved the most similar report by similarity comparisons. Alksas et al. [27] utilized Random Forest to classify the liver tumor grades of LR1, LR2, LR3, LR4, and LR5.

### C. Liver Partition

**FIGURE 2.**
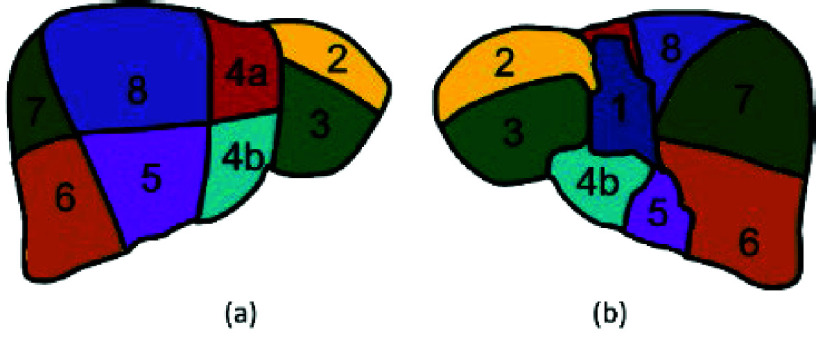
Couinaud segments in (a): anterior view and (b): posterior view.

Typically, the liver can be divided into 8 functionally independent regions called Couinaud Segments by the portal vein, as shown in Figure 2. In general, at least 2 Couinaud segments will be reserved after a liver hepatectomy due to concerns of segment remnant functions. Hence, preoperative assessment for hepatic functional reserve is an important issue. Hernandez-Alejandro et al. [28] proposed a proposal for staged liver resections by bridging couinaud segments to portal vein ligation. Both Lebre et al. [29] and Oliveira et al. [30] performed liver partition by stages. First, the liver was segmented. Then, the portal veins were identified, and finally, the liver was partitioned into 8 segments by the portal veins. Han et al. [31] used a single 3D Unet to directly segment liver segments from MRI images. In contrast to the reference [31], this paper proposes multi-classifiers from viewpoints of different segments, and further conducts the better results for both 2D and 3D images.

## III. Proposed Framework for Liver-Tumor Annotation

### A. Framework

**FIGURE 3.**
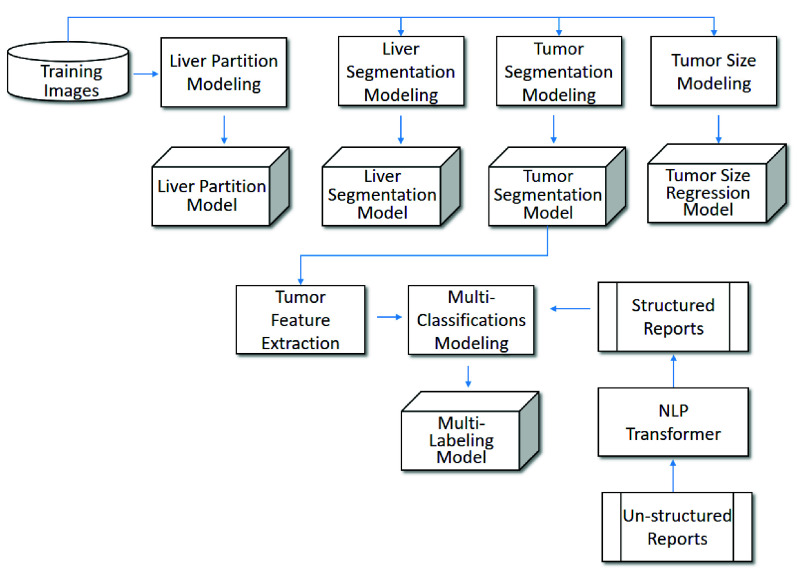
Offline training of proposed system framework.

**FIGURE 4.**
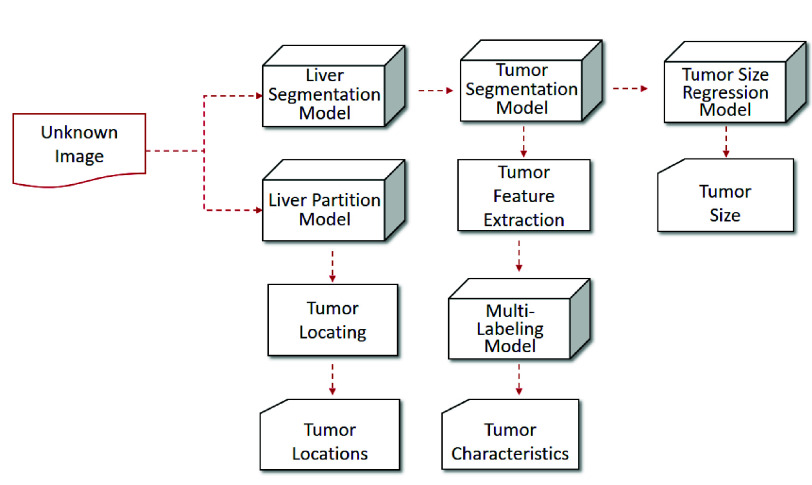
Online annotation of proposed system framework.

To achieve high quality of tumor annotation, Figures 3 and 4 show the framework of proposed system comprising offline training and online annotation.

• Offline training (illustrated in Figure 3): This stage trains models for four main components. In terms of tumor segmentation, it needs a liver segmentation model and a tumor segmentation model. In terms of tumor recognition, it needs a multi-labeling model combining information of tumor features and structured reports labels. In terms of tumor measuring, it needs a tumor size regression model. In terms of tumor location, it needs a liver partition model.
• Online annotation (illustrated in Figure 4): On the basis of the models trained, first, the liver and tumor are sequentially segmented from the unknown image. That is, the whole liver is split from the image and then the tumor is segmented from the liver. Next, the liver is partitioned into 8 regions and the tumor will be located accordingly. Simultaneously, the tumor features are extracted for multi-labeling the tumor. Also, the tumor size is predicted by the regression model. Finally, the tumor size, tumor location and tumor characteristics can be incorporated as annotations into the diagnosis report.

### B. Offline Training

#### 1) Generating Ground Truths from Unstructured Reports

This is an essential operation which can be viewed as knowledge discovery. Since existing diagnostic reports are expressed in natural language, it is necessary to transform these unstructured sentences into structured labels. For this need, the unstructured reports are transformed into the labels as ground truths. Because this operation is not the primary focus of this paper, we just utilized the previous work [2] to perform it, achieving an accuracy of approximately 90.99%.

#### 2) Tumor Segmentation

**FIGURE 5.**
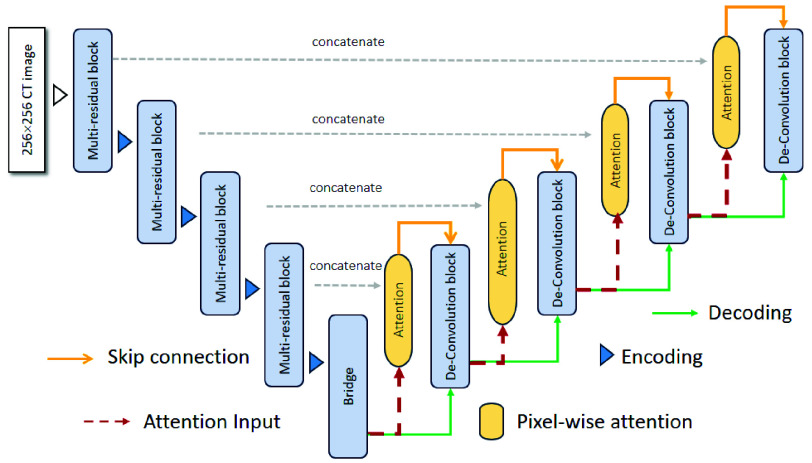
Architecture of proposed multi-residual attention Unet.

In our proposed method, the tumor segmentation consists of two steps, namely liver segmentation and tumor segmentation. Thus, it needs two segmentation models for livers and tumors, respectively. For modeling the liver segmentation, it is implemented by Unet [9] with data augmentations of flipping. Finally, the dice of the liver segmentation reached 0.951. For modeling the tumor segmentation, Figure 5 shows the architecture of the proposed Multi-Residual Attention Unet consisting of an encoder and a decoder. The encoder is composed of 4 multi-residual blocks, while the decoder is composed of 4 de-convolution blocks. In the followings, the main ideas of multi-residual blocks, pixel-wise attention and loss functions will be presented in detail.

### C. Multi-Residual Block

In this component, the major intent is to cope with the issues of *information loss* and *uniform feature weighting* by leveraging two key concepts: multiple residuals and attention mechanisms. In terms of *information loss*, information in traditional deep networks could be lost through multiple convolutional layers. To address this issue, we employ multiple continuous residual connections to preserve and enhance information during the convolution process. In terms of *uniform feature weighting*, traditional models process input features uniformly, making it difficult to emphasize the more important parts. To aim at this issue, we employ the attention mechanism [33] to dynamically learn the importance of each input position, thereby improving model performance. In overall, Multi-residual Block takes a dual mechanism of dynamic weighting and information enhancement, enabling it to effectively handle the challenges of tumor segmentation.

**FIGURE 6.**
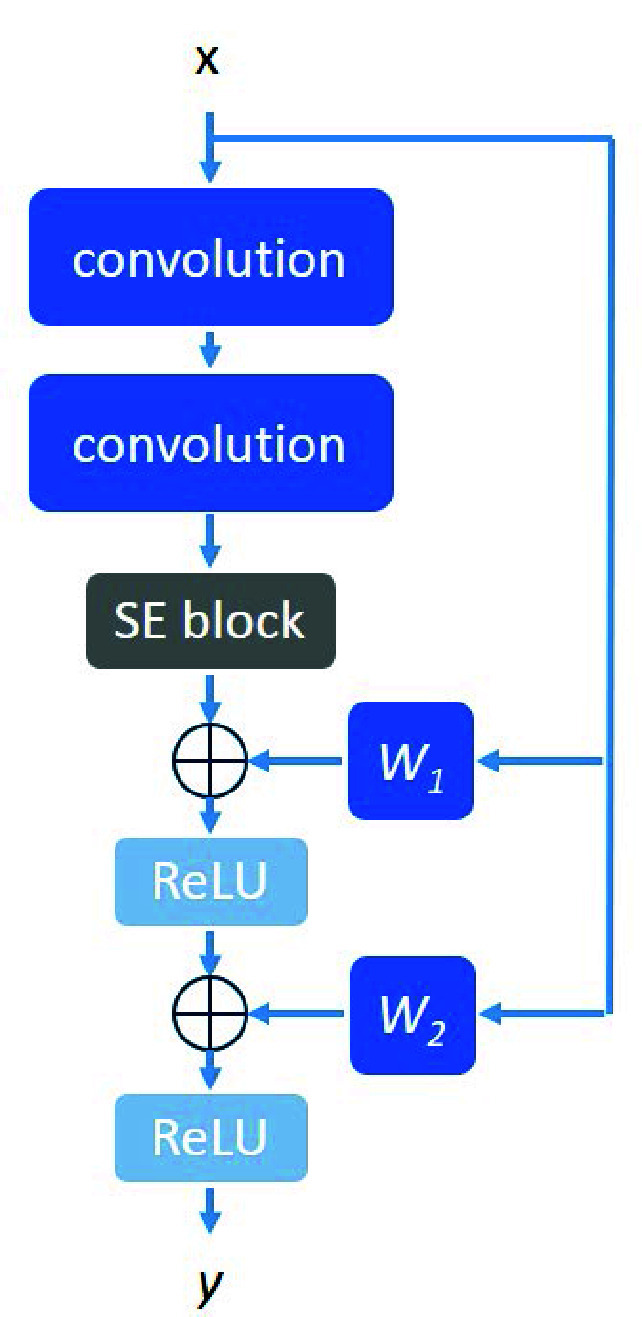
Architecture of proposed multi-residual block.

Figure 6 shows the architecture of the proposed multi-residual block, consisting an input *x*, an output *y*, two convolutions, a Squeeze-and-Excitation block (called SE block) [34], two activation functions ReLU and two weight functions $W_{1}$ and $W_{2}$. Here, $W_{1}$ and $W_{2}$ are the linear mapping functions using the $1\times 1$ convolutions to achieve the linear transformations. For the squeeze-and-excitation block, it can actually be regarded as a channel-wise attention mechanism consisting of two main modules. The first module Squeeze is set for aggregating the features. The formula for aggregations is defined as:

\begin{equation*} z_{c}=\frac {1}{h\mathrm {\times }w}\sum \limits _{i=1}^{h} \sum \limits _{j=1}^{w} {u_{c}(i,j)}' \tag {1}\end{equation*}

where *z* denotes a scalar for the attention coefficient, *c* denotes the channel number, *h* and *w* denote height and width, respectively, and *u* denotes the aggregation function for pixels of *i* and *j* in a channel. The major idea of this formula is to apply Global Average Pooling to each channel (i.e., averaging over the entire spatial dimensions $h\times w$) to obtain a single value per channel. This effectively compresses each channel’s feature map into one scalar, forming a vector of size $c\times 1$. In this step, all pixels in the spatial dimension are averaged, indicating that the pixels are not independent but they collectively contribute to the overall representation of the channel. After aggregations, the second module Excitation is set to extract attention features from the aggregated information, generating the attention coefficient. The formula for excitation is defined as:

\begin{equation*} s=\sigma (F_{2}\delta (F_{1}z)), \tag {2}\end{equation*}

where *z* stands for the feature vector derived by Equation (1), $F_{\mathrm {1}}$and $F_{2}$ are the linear transformation functions used in the fully connected layer, $\delta $ and $\sigma $ stand for the ReLU activation function and the sigmoid activation function, respectively. Therefore, the attention coefficient derived by Equation (2) can be employed to enhance the model segmentation quality. In summary, through our proposed multi-sequential weighted identity mapping, the feature information can be enhanced. That is, the potentially interested features can be focused by the squeeze-and-excitation block. This is the main uniqueness of the proposed multi-residual block.

### D. Pixel-Wise Attention

**FIGURE 7.**
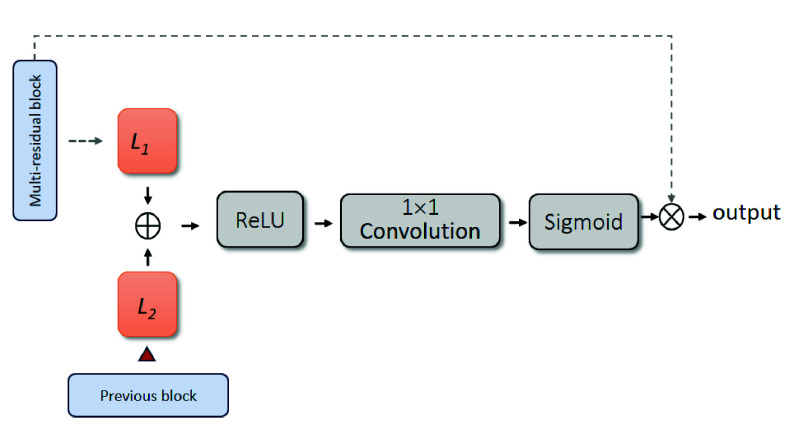
Workflow of pixel-wise attention.

In order to make the segmentation focus on the tumor more, the pixel-wise attention mechanism [13] (also called Spatial-wise attention) is embedded into the decoder. Figure 7 illustrates the pixel-wise attention mechanism. In this framework, L1 and L2 are two linear transformation functions implemented using $3\times 3$ convolutions, applied respectively to the two inputs of the pixel-wise attention module. Specifically, L1 applies a $3\times 3$ convolution to the input feature map from the multi-residual block to obtain the first transformed feature map, while L2 applies a $3\times 3$ convolution to the input feature map from the previous block to obtain the second transformed feature map. Next, the addition of two feature maps is passed through a RelU function, followed by a $1\times 1$ convolution and a sigmoid function. Finally, these weights are element-wise multiplied with the original multi-residual block. As a result, the whole segmentation network is guided to focus more effectively on the target object.

### E. Loss Coefficient

The final idea to present in Multi-Residual Attention Unet is the used loss coefficients, including Dice Coefficient, Binary Cross-entropy Coefficient and Matthews Correlation Coefficient. The dice coefficient [35] is a popular metric widely used in evaluating image segmentation quality, indicating the intersection ratio between the prediction and the ground truth. The Binary Cross-Entropy *BCE* is adopted to measure the object classification quality in this paper, which is defined as:

\begin{equation*} BCE=-(\left ({{ 1-y }}\right )\ast log(1-p)+y\times log\left ({{ p }}\right )), \tag {3}\end{equation*}

where *y* and *p* denote the true binary and the probability of the predicted positive class, respectively. The Matthews correlation coefficient *MCC* [36] is a binary measurement for classifications, measuring the relationships among False Positives (FP), True Positives (TP), False Negatives (FN), and True Negatives (TN) in a confusion matrix. It is defined as:

\begin{equation*} MCC={MCC}_{n}/\sqrt {MCC}_{d}, \tag {4}\end{equation*}

where ${MCC}_{d}=\left ({{ TP+FN }}\right)\ast \left ({{ TP+FP }}\right)\ast (TN+FN)\ast \left ({{ TN+FP }}\right)$ and ${MCC}_{n}=(TP\ast TN)-\left ({{ FP\ast FN }}\right)$.

#### 1) Tumor Location

**FIGURE 8.**
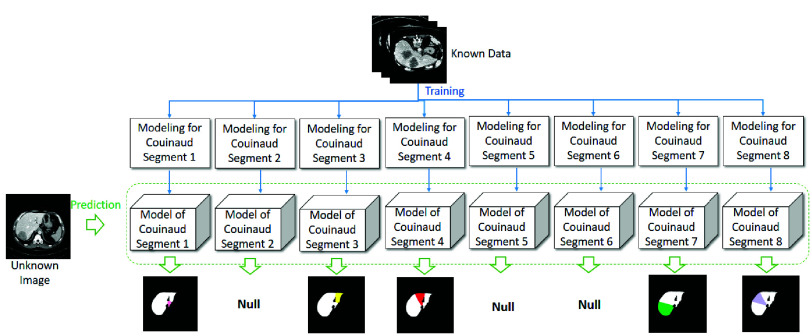
Architecture of proposed Multi-SeResUnet.

In the online stage, as shown in Figure 8, it needs to partition the liver into 8 couinaud segments before locating the tumor. Therefore, in the training, the liver partition model has to be constructed. In this paper, innovative segmentation methods for 2D and 3D images are proposed where the core creativity is to reduce the segmentation complexity. Traditional segmentation methods attempt to partition the objects by a single segmentor but the effectiveness is limited. In our approach, 8 segmentors are conducted individually. That is, the computer vision will be limited in the smaller space and therefore the effectiveness will be increased obviously.

For 2D images, the proposed method, called Multi-SeResUnet, utilizes SeResUnet [4] to construct eight recognition models. The detailed framework can be found in the related work [37]. For a 3D image [31], [38], which contains 3D kernels used for convolutions and max-pooling operations. Except for the final convolution, the kernel with size of $3\times 3$ is used for the other convolutions. Also, the batch normalization (BN) along with the LeakyReLU activation function is used for these convolutions. The transposed convolutional layers and max pooling operations use a kernel size $2\times 2$. The final convolutional layer generates the output, while the related kernel size is $1\times 1$ and the related activation function is sigmoid. Whether the image is 2D or 3D, the major uniqueness for liver partition in this paper is to split a single partition model into 8 individual partition models to reduce the partition complexity. That is, for each of couinaud segments, a referred model is trained.

#### 2) Tumor Measuring

Tumor size is an important evidence for stage diagnosis, surgical treatment and prognosis estimation. In general, the tumor size ranges from 0.5cm to more than 20cm, with the majority being less than 5cm, followed by 5-10cm. Few tumors will grow to more than 10cm. Currently, treatments for liver tumors below 3cm are relatively selective, and the prognosis is relatively good. This is why the aimed annotation contains tumor size. Because the tumor is segmented in a visual format of pixels, it needs a mechanism to transform the pixels into centimeters. Hence, the size here is approximated by a linear regression function, which is defined as:

\begin{equation*} {\it size} = b + a \times {\it length}, \tag {5}\end{equation*}

where *b* stands for the intercept, *a* stands for the regression coefficient and *length* stands for the longest Euclidean distance between the tumor border points. In this paper, *b* and *a* are set as -0.2011 and 0.0748, respectively.

#### 3) Tumor Recognition

In this work, tumor recognition refers to the multi-labeling algorithm learning from visual features on recognizing the tumor characteristics. For this purpose, the *Radiomics* features [39], [40] are selected as the visual features. In this stage, the *Radiomics* features are extracted first. Next, a set of nearly optimal features are filtered by the feature selector. Finally, the multi-labeling classifier is performed to recognize the potential characteristics. In order to pursue a high-quality classification, sets of feature selectors and multi-labeling classifiers were evaluated in the experiments. Specifically, feature selectors such as Information Gain and Pearson Correlation Coefficients, and classifiers including Support Vector Machine (SVM), Linear Discriminant Analysis (LDA), Random Forest (RF), Neural Network (NN), AdaBoost, and XGBoost were assessed.

### F. Online Annotation

On the basis of the above training models, the automated annotation starts with a submission of an unknown CT image. First, the liver is segmented by Unet. Second, the tumor is initially split from the liver by Multi-Residual Attention Unet. Because the initial tumor segmentation is not very solid, it needs an additional post-process. Here, the post-process comprises two further operations, namely thresholding and morphological operations. Through thresholding, the most potential pixels will be determined from the initial tumor segmentation, while morphological operations such as closing and opening make the tumor segmentation more solid. In particular, morphological operations are performed in three dimensions in a CT image cube, with respect to xy, xz and yz planes. Note that, the threshold is set as 2 in this paper. Simultaneously, the liver is partitioned by Multi-SeResUnet. Third, the *Radiomics* features are extracted from the tumor and the corresponding characteristics are subsequently classified. Fourth, the tumor is measured by Equation (5). Fifth, the tumor is located by checking every couinaud segment with Equation (6) which is defined as:

\begin{align*} & location\left ({{ s_{k} }}\right ) \\ & \!\!=\!\!\begin{cases} \displaystyle true,& if \frac {predicted \cap ground\_truth}{ground\_truth} \!> \! 0.25 \\ \displaystyle false,& otherwise \end{cases}, \tag {6}\end{align*}

where $s_{k}$ indicates the $k^{\mathrm {th}}$ couinaud segment label, *predicted* indicates the partitioned couinaud segment, and *ground_truth* indicates the actual couinaud segment. That is, if $location\left ({{ s_{k} }}\right)$ is true, the resulting locations will include $s_{k}$. Finally, the tumor characteristics, tumor size and tumor location are merged into the final diagnosis report.

## IV. Experiments

### A. Experimental Settings

#### 1) Experimental Data

The overall experiments were conducted on an aspect of evaluating the proposed components, including tumor segmentation, tumor location, tumor measuring and tumor recognition. Therefore, three datasets were set for evaluating these components. In terms of tumor segmentation, Lits data [41] were set as the experimental data, including 131 patients. In terms of tumor location, it needs to partition the liver in advance. For this need, MedSeg data [42] were set for liver partition, including 50 patients. In terms of tumor measuring and tumor recognition, the experimental data were gathered from the hospital in Taiwan, including 70 patients. The number of labels for tumor recognition is 11. In the related evaluations, 5-fold cross validations were set for tumor segmentation, liver partition and tumor recognition, while tumor measuring was experimented by a 2-fold cross validation. Note that, the proposed evaluation shows the robustness and generalization of proposed system because the evaluation was conducted using models constructed from three different datasets. The LiTS dataset was used to build the liver segmentation model. Based on its results, the Chang Gung dataset was employed for tumor segmentation model construction. Additionally, the MedSeg dataset was used to develop the liver partition model. Finally, tumors in the Chang Gung dataset were located and annotated by integrating the results of tumor segmentation and liver partition.

#### 2) Experimental Network Parameters

The involved Unets in this paper include Multi-Residual Attention Unet, SeResUnet and 3D Unet. Table 1 shows the detailed settings of experimental network parameters.

**TABLE 1:** Settings of Experimental Network Parameters

Network	batch size	resize	epoch	optimizer	learning rate
Multi-Residual Attention Unet	16	$256\times 256$	50	Adam	0.0001
SeResUnet	8	$256\times 256$	50	Adam	0.0001
3D Unet	2	$64\times 128 \times 128$	80	Adam	0.0001

#### 3) Experimental Measures

The measures used in the evaluations can be divided into 3 categories for segmentation/partition, measuring and recognition/location, respectively. For segmentation and partition, dice is the main metric revealing the Harmonic mean between True Positive Rate and Positive Predictive Value. For measuring, MAE (Mean Absolute Error) and RMSE (Root Mean Squared Error) are two related metrics. For recognition and location, Accuracy, Precision, Recall and F1*-*score widely used in machine learning evaluations are four main metrics.

### B. Evaluations Results

#### 1) Experimental Results for Tumor Segmentation

The experimental results for tumor segmentation are analyzed in two main aspects: approximation of settings and overall performance comparison. For approximation of settings, the better settings for #residuals, attention mechanisms, loss functions and morphological operations are elicited before overall comparisons. Next, the proposed method was compared with a set of state-of-the-art methods.

Table 2 lists the compared methods with terminologies. Because there have been so many recent works accompanying Unets with InceptionNet [43] and RseNet [32], we do not list the specific cited references here.

**TABLE 2:** Terminologies of Involved Methods Compared in the Experiments

Aspect	Compared Method	Terminology
Tumor Segmentation	Unet [9]	Unet
	Attention Unet [13]	AUnet
	Unet with Backbone ResNet	RUnet
	SE-Unet [34]	SeUnet
	Unet++ [11]	Unet++
	Unet with residuals and Backbone InceptionNet v3	IUnet
	MRAUnet	proposed
Feature Selection	Pearson Correlation Coefficient	PCC
	Information Gain	IG
Tumor Recognition	Support Vector Machine	SVM
	Linear Discriminant Analysis	LDA
	Random Forest	RF
	Neural Network	NN
	AdaBoost	AdB
	XGBoost	XGB

**FIGURE 9.**
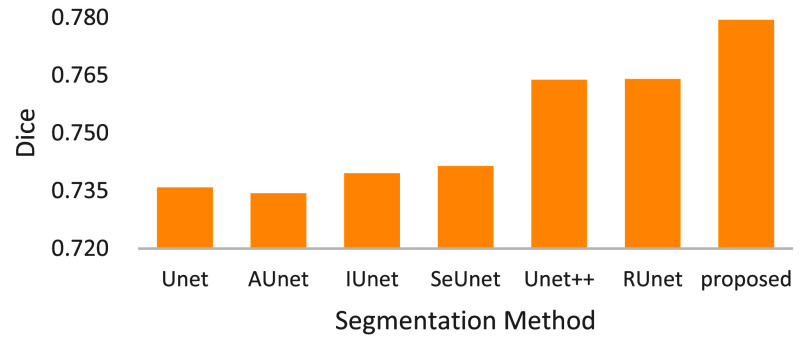
Dices of compared tumor segmentation methods.

Figure 9 depicts the dices of compared tumor segmentation methods, delivering several observations. First, the results can briefly be clustered into 4 groups, namely {Unet, AUnet}, {IUnet, SeUnet}, {Unet++, ResUnet} and {proposed}, where the performance rank is {proposed}, {Unet++, ResUnet}, {IUnet, SeUnet} and {Unet, AUnet}. In the results, the potential reason for why Unet and AUnet perform closely could be that, the bridge attention does not deal with small tumors very well. Second, pure residual-based Unets such as Unet++ and ResUnet perform better than the others except the proposed method. Third, the proposed MRAUnet achieves the best dice. From above results, the technical contribution can be echoed with a concept that, the proposed MRAUnet with multiple sequential residuals and attention mechanisms brings out the better segmentation quality than the compared methods.

#### 2) Experimental Results for Tumor Location

**FIGURE 10.**
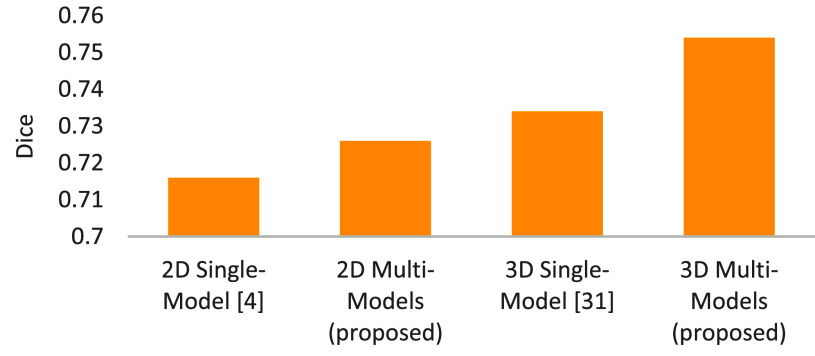
Dices of liver partition using multi-models and single models under 2D and 3D spaces.

**FIGURE 11.**
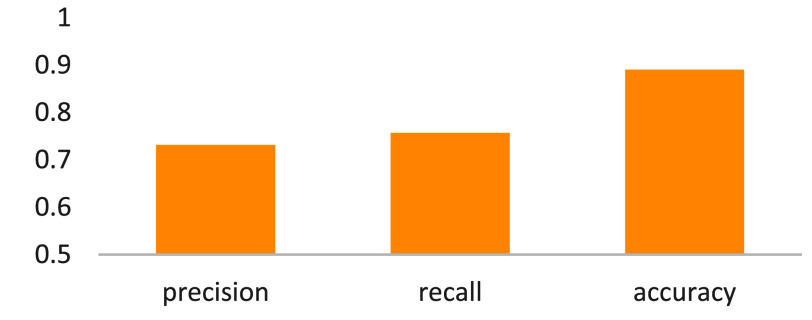
Precision, recall and accuracy of tumor location using 2D multi-models.

The tumor location was implemented by two phases, including liver partition and tumor location. In terms of liver partition, we compared the proposed multi-models with the related single models [4], [31]. The liver-partition dices in 2D and 3D spaces are shown in Figure 10. From this result, we can obtain two observations that, first, 3D models outperform 2D models. Second, the proposed multi-models work better than the compared single models. Here, this result can be viewed as one of our technical contributions. Following this, we will look into the tumor location. On the basis of liver-partition results, the evaluation results for tumor location are shown in Figure 11. The precision, recall, and accuracy were found to be satisfactory by the doctors after reviewing the results.

#### 3) Experimental Results for Tumor Measuring

Tumor measuring is proposed to predict the tumor size by Equitation (5). Table 3 shows the tumor size distribution. Most tumor sizes range from 1 to 5 centimeters. Table 4 reveals the evaluation results in terms of MAE and RMSE, which can be explained by some concepts. First, the averaged MAE is much smaller than size of 5cm, around an error ratio of 0.0866cm. Second, in contrast to MAE, RMSE is slightly higher than MAE. This is because RMSE is more sensitive to the larger prediction losses. Overall, the results show the proposed method is stable. For robustness concern, it is further analyzed statistically. Table 5 depicts that the proposed tumor measuring is robust while the PCC and p value achieve 95.15% and 0.000, respectively. For PCC, it indicates the prediction results are highly related to the ground truths. For p value, it is 0.000 smaller than the threshold of 0.01, revealing there exists an obvious relevance between prediction results and ground truths. These evaluation results evidence the robustness of the proposed tumor measuring.

**TABLE 3:** Tumor Size Distribution

	<1cm	1-5cm	6-10cm	>10cm
#tumors	1	61	7	1

**TABLE 4:** Experimental Results for Tumor Measuring

Metric	Average
MAE	0.432996
RMSE	0.638784

**TABLE 5:** Statistical Analysis for Tumor Measuring

Metric	value
PCC	0.951458
p value (<0.01)	0.000

#### 4) Experimental Results for Tumor Recognition

**FIGURE 12.**
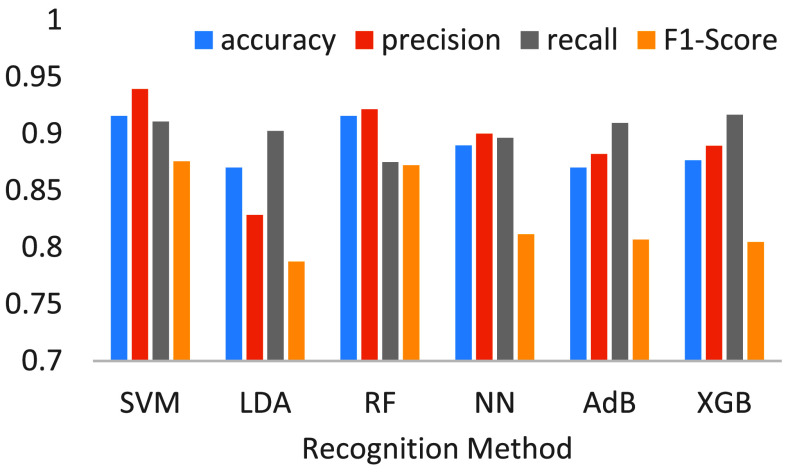
Effectiveness comparisons for tumor recognition models.

Figure 12 shows the comparative results for tumor recognition. In this evaluation, 1316 visual features are extracted from a tumor. Here, several observations are summarized. First, in overall, SVM is the best, and RF is the second top whether using IG or PCC. However, the performances of SVM and RF are pretty close. This could be explained by the fact that, SVM uses a nonlinear kernel function, while RF inherently exhibits nonlinear characteristics. Both models can effectively handle the nonlinear data in this experiment. On average, SVM is more stable than RF due to its higher precision and recall. Second, LDA performs the worst among the compared methods. The potential reason could be that, LDA is based on idealized linearity assumptions of linearity and Gaussian distribution. Because the experimental data in this study do not meet these assumptions, LDA naturally performs worse than more flexible, nonlinear, or ensemble-based models. In contrast, RF and XGB are ensemble learning models capable of constructing complex and flexible decision boundaries. NN can learn deep features and handle high-dimensional and nonlinear data. AdB adapts by focusing more on the misclassified samples. Third, although the precisions and recalls of NN, AdB and XGB are not low, their F1-scores are lower than those of SVM and RF. This might be caused by the fact that, F1-score is calculated by first taking the harmonic mean of precision and recall for each individual record, and then averaging the results. It penalizes cases where there is a large discrepancy between precision and recall. Therefore, if the model’s performance across records is diverse, it can significantly lower the overall F1-score. In other words, the precisions and recalls of NN, AdB and XGB show significant instability, with substantial variability in individual records. In summary, SVM and RF are two notable classifiers recommended in this paper due to their consistently high and closely matched performances. Note that, for feature selections, IG is slightly better than PCC on the whole. To reveal the model interpretability, Table 6 presents the top 10 Radiomics features used in the recognition models, highlighting that shape and first-order statistical features are the most influential. Shape features describe the geometric properties of tumors, such as volume, surface area, aspect ratio, roundness, and boundary irregularity. In contrast, first-order statistics are derived from the distribution of gray-level intensities within the image, without accounting for the spatial relationships between pixels.

**TABLE 6:** Top 10 Radiomics Features

Rank	Feature
1	original_shape_Elongation
2	original_shape_MajorAxisLength
3	original_shape_MeshVolume
4	original_shape_MinorAxisLength
5	original_shape_Sphericity
6	original_shape_SurfaceArea
7	original_shape_SurfaceVolumeRatio
8	original_firstorder_Energy
9	original_firstorder_Entropy
10	original_firstorder_Kurtosis

## V. Conclusion

Recent Bioinformatics techniques have been effectively applied to liver tumor segmentation over the past few years. Yet, there remain some issues in these methods. First, their effectiveness could be improved. Second, no study has proposed a comprehensive tumor annotation system. To address these issues, in this paper, we propose a set of effective methods to achieve an automated tumor annotation system for diagnosis. First, the annotation format is defined by the doctors based on practical requirements. Second, unstructured reports are transformed into structured labels for training. Third, tumor segmentation is performed using the proposed MRAUnet, which incorporates Unet, SE-Bolck, pixel-wise attention, channel-wise attention and post-processing. Fourth, the tumor is located within the specific Couinaud segments, which are partitioned by the proposed Multi-SeResUnet. Fifth, the tumor is measured by the proposed regression model. Sixth, the tumor characteristics are classified by the approximated multi-labeling method. Finally, the location, size and characteristics of the tumors are viewed as annotations merged into the diagnosis report. To assess the effectiveness of proposed methods, a number of evaluations were conducted on real datasets. The evaluation results reveal that the proposed methods indeed yield satisfactory outcomes for radiologists. Accordingly, this approved annotation system will be integrated into the existing medical infrastructure to assist radiologists in generating effective reports more efficiently and thereby help prevent treatment delays.

In the future, several issues need to be addressed. First, a general system using different datasets is demanded by the doctors because the support hospital has different branches with different CT capturing devices. Federated learning and collaborative learning are the next intents in our future work. Second, the effectiveness is related to the GPU performances, making the model un-afford big data. Hence, the data augmentation was not used in the tumor segmentation and liver partition. In the future, we will make further training models with data augmentations by the greater GPU. Third, for liver partition, 3D Unet is a potential model but its performance was limited in GPU performances. Therefore, the better version will be researched in the future by using the more powerful GPU. Fourth, the location, size and characteristics are the aimed attributes in this paper. In addition to these attributes, future work will incorporate more tumor information, such as stage, invasion, recurrence, and others, into the annotation system to enhance its robustness.

## Author Contributions

Conceptualization: Yi-Hsuan Chuang, Ja-Hwung Su, Yi-Wen Liao, Yeong-Chyi Lee, and Yu-Fan Cheng; Methodology: Yi-Hsuan Chuang, Ja-Hwung Su, Tzung-Pei Hong, and Katherine Shu-Min Li; Validation: Tzu-Chieh Lin, Hue-Xin Cheng, Pin-Hao Shen, Jin-Ping Ou, and Ding-Hong Han; Formal Analysis: Ja-Hwung Su, Yi-Wen Liao, and Yeong-Chyi Lee; Investigation: Yu-Fan Cheng, Tzung-Pei Hong, Katherine Shu-Min Li, and Chih-Chi Wang; Data Curation: Yi-Hsuan Chuang, Yu-Fan Cheng, Yi Lu, and Chih-Chi Wang; Resources: Yi-Hsuan Chuang, Yu-Fan Cheng, Yi Lu, and Chih-Chi Wang; Writing—Original Draft Preparation: Ja-Hwung Su; and Writing—Review and Editing: Ja-Hwung Su.

## Institutional Review Board Statement

The experimental data were approved by Kaohsiung Chang Gung Memorial Hospital, Taiwan. The approval information for Institutional Review Board is shown as follows. -Ethic Committee Name: Chang Gung Medical Foundation -Approval Codes: IRB No.: 202100262B0 and 202300484B0 -Approval Dates: 2021/04/14 and 2023/04/27. All operations in this paper were executed according to the ethical standards of the Institutional Review Board, Taiwan.

## Conflict of Interest Disclosure

The authors declare no conflict of interest.

## Patient Consent Statement

Because this is a retrospective study without personal identification information, we did not use the informed consents.

## Clinical Trial Registration

This is a retrospective study that does not involve any clinical trial as defined by the World Health Organization or other regulatory bodies. Therefore, it was not registered in any clinical trial registry.
